# Maternal Dietary Quality and Dietary Inflammation Associations with Offspring Growth, Placental Development, and DNA Methylation

**DOI:** 10.3390/nu13093130

**Published:** 2021-09-08

**Authors:** Marion Lecorguillé, Shevaun Teo, Catherine M. Phillips

**Affiliations:** School of Public Health, Physiotherapy and Sports Science, University College Dublin, Dublin 4, Ireland; shevaun.teo@ucdconnect.ie (S.T.); catherine.phillips@ucd.ie (C.M.P.)

**Keywords:** maternal dietary quality, dietary inflammatory index (DII), dietary scores, placental development, birth outcomes, childhood obesity, epigenetics

## Abstract

The ‘Developmental Origins of Health and Diseases’ hypothesis posits that prenatal maternal diet influences offspring growth and later life health outcomes. Dietary assessment has focused on selected nutrients. However, this approach does not consider the complex interactions between foods and nutrients. To provide a more comprehensive approach to public health, dietary indices have been developed to assess dietary quality, dietary inflammation and risk factors for non-communicable diseases. Thus far, their use in the context of placental development is limited and associations with offspring outcomes have been inconsistent. Although epidemiological studies have focused on the role of maternal diet on foetal programming, the underlying mechanisms are still poorly understood. Some evidence suggests these associations may be driven by placental and epigenetic changes. In this narrative review, we examine the current literature regarding relationships between key validated diet quality scores (Dietary Inflammatory Index [DII], Mediterranean diet [MD], Healthy Eating Index [HEI], Alternative Healthy Eating Index [AHEI], Dietary Approaches to Stop Hypertension [DASH], Glycaemic Index [GI] and Glycaemic Load [GL]) in pregnancy and birth and long-term offspring outcomes. We summarise findings, discuss potential underlying placental and epigenetic mechanisms, in particular DNA methylation, and highlight the need for further research and public health strategies that incorporate diet quality and epigenetics.

## 1. Introduction

The importance of adequate nutrition during foetal life for long-term health is well documented [1]. The ‘Developmental Origins of Health and Diseases’ (DOHaD) hypothesis postulates that maternal nutritional status in the preconception period and during pregnancy may influence offspring development and the occurrence of disease, from childhood to later life [2,3,4,5]. More recently, research in DOHAD shows that environmental factors can affect the development of the next generation even before conception and into early childhood (the first 1000 days—from conception to two years). The Dutch famine studies have reported that women who were undernourished during pregnancy had increased risk of having children with intrauterine growth retardation (IUGR) [2]. Smaller size or relative thinness at birth or during infancy has been related to increased rates of cardiovascular disease (CVD), type 2 diabetes mellitus (T2DM) and metabolic syndrome (MetS) in adulthood [5,6,7,8]. Much attention has focused on the conditions of nutritional deprivation, but there is new evidence that maternal obesity during pregnancy is associated with long-term health consequences for the offspring, such as increased body mass index (BMI) during infancy, childhood and later life and increased risk of T2DM in adulthood [9,10].

Adequate maternal nutritional intake is an essential factor for a healthy pregnancy and optimal child development. However, dietary assessment is difficult to elucidate due to its complex interactions between diverse foods and nutrients [11]. To provide a more comprehensive approach to public health prevention, several aspects of dietary intake are measured against dietary recommendations (defined as ‘dietary quality’) [12,13]. Pregnancy is also known to alter the maternal inflammatory state, and unfavourable levels of pro-inflammatory cytokines may be in turn associated with pregnancy complications and adverse birth outcomes [14]. Diet has been recognised as an important modulator of chronic inflammation. Furthermore, having a more anti-inflammatory or less pro-inflammatory diet may reduce adverse outcomes during pregnancy, particularly in obese women [14,15].

Although epidemiological studies have focused on the role of maternal diet on foetal programming, underlying mechanisms are still poorly understood. Perturbations in the maternal environment may be transmitted across the placenta and thereby affect the development of the foetus. The placenta plays a fundamental role in maternal-foetal exchanges during pregnancy and an adequate maternal nutritional status is necessary to support placental homeostasis and development [16]. Altered placental structure and function may contribute to altered nutrient supply to the foetus [17].

Some evidence has shown that these associations may be also driven by epigenetic changes [18]. Epigenetic marks, in particular DNA methylation, appear to be particularly sensitive to environmental exposures during embryogenesis and cell differentiation [18,19]. Epigenetic data in humans are becoming increasingly robust but there is a challenge to continue exploring how specific factors (particularly nutritional factors in the prenatal period) may affect epigenetic mechanisms.

Thus, the aim of this narrative review is to examine and synthesize the literature regarding associations between maternal dietary quality, evaluated by several dietary scores, and dietary inflammation state with birth outcomes and long-term offspring health. We examine the underlying mechanisms including the placental dysfunction, which in turns impacts foetal growth, and the epigenetic mechanisms, more specifically DNA methylation, and conclude with future perspectives in this area of nutritional research

## 2. Materials

This is a narrative review of the current literature on the relationships between maternal dietary quality and dietary inflammation status and, birth and child outcomes, placental development and epigenetics. The literature search was performed in Pubmed, Web of Science, Scopus and the Cochrane database by using the following keywords separately or in combination: Maternal diet, nutrition, dietary inflammatory index, dietary quality scores (Dietary Inflammatory Index [DII], Mediterranean diet [MD], Dietary Approaches to Stop Hypertension [DASH], Healthy Eating Index [HEI], Alternative Healthy Eating Index [AHEI], Glycaemic Index [GI], Glycaemic Load [GL]), macronutrients, micronutrients, pregnancy, periconception period, foetal programming, child development, birth anthropometry, obesity, body mass index, placenta, epigenetic mechanisms, DNA methylation and cord blood. Relevant free-access abstracts were identified and peer-reviewed human studies, meta-analyses, systematic reviews and interventional studies were included. Other inclusion criteria were: (1) English language; (2) recording of the diet before or during pregnancy; (3) assessment of offspring measurements and prematurity; and (4) published papers within the last 15 years. Additionally, we included animal studies in our literature search in relation to maternal diet associations with placental outcomes and epigenetics. The websites of key government departments and agencies were consulted to identify important recommendations and reports such as the “Dietary Guidelines for Americans 2010”.

## 3. Maternal Diet and Indices of Dietary Quality and Dietary Inflammation

Optimisation of maternal diet during critical windows of development may positively impact on childhood weight status and future health trajectory [3,20]. However, the specific dietary requirements during pregnancy for optimal foetal growth and development are often limited in applicability and often prioritise macronutrient and micronutrient intake rather than food groups and dietary patterns. Most of the randomised controlled trials (RCTs) and observational studies have evaluated individual nutrient effects on foetal growth, with particular emphasis on avoiding certain micronutrient deficiencies [21,22]. Dietary patterns may provide a more comprehensive approach in public health for mother and infant as they have the potential to be used as a valid tool in assessing dietary associations with pregnancy outcomes [11,23]. Overall dietary quality may be more translatable to public health guidelines. Therefore, a variety of dietary scores and indices have been developed for nutritional epidemiology to assess dietary risk factors for non-communicable diseases [24,25,26,27]. These algorithms aim to evaluate the overall diet and categorise individuals according to the extent to which an individual’s eating behaviour is “healthy” based on primary nutritional data collected from 24-h quantitative dietary intake recalls, dietary records and food frequency questionnaires (FFQs) [28]. More recently, the dietary inflammatory index (DII^®^), was developed to characterise the inflammatory potential of the diet [25,29,30,31]. Here we describe dietary components, patterns and scores that reflect dietary quality and inflammation and their relevance in pregnancy.

### 3.1. Macronutrient Intakes in Pregnancy

Macronutrients are broadly defined as nutrients that provide calories or energy and are required in large amounts to maintain body functions and carry out the activities of daily life [32,33]. During pregnancy, the maternal diet must provide an adequate supply of energy to support the mother’s usual requirements as well as those of the growing foetus [21]. Extra energy is required for the synthesis of new tissue (foetus, placenta and amniotic fluid) and the growth of existing tissue (uterus, breast and maternal adipose tissue). Excessive energy intake should be avoided as it is the main determinant of gestational weight gain [21]. Hence, balancing and optimising protein and fibre as macronutrients could help with avoiding overnutrition during pregnancy, but these proportions need to be tailored based on pre-pregnancy weight, ethnicity, health literacy, BMI status and stage of pregnancy [21,33,34]. Special attention is warranted for the impact of carbohydrate intake in those with poor glycaemic control and at risk of gestational diabetes mellitus (GDM). During pregnancy, glucose transfer mainly occurs in the postprandial state, and glucose concentrations are determined by the carbohydrate foods [35,36]. The glycaemic index (GI) and glycaemic load (GL) qualify and quantify the postprandial glycaemic responses according to the dietary carbohydrate intake. Carbohydrates with low GI, for example whole grain breads, cereals and nuts, lead to a low glycaemic response, whereas foods such as confectionary and soft drinks lead to high glycaemic response [36,37]. Many systematic reviews have established the links between dietary GI/GL and the risk of chronic disease (such as T2DM, CVD and various cancers) but maternal carbohydrate quality has received relatively little attention [38,39]. Hence, further research is required in establishing the optimum timing to start a low-glycaemic index (GI) or glycaemic load (GL) diet for maximum protection against adverse pregnancy outcomes, in terms of insulin sensitivity, for both mother and infant [35,40].

### 3.2. Micronutrient Intakes in Pregnancy

Micronutrients are vitamins and minerals needed by the body in very small amounts to enable production of enzymes, hormones and other substances needed for normal growth and development [41,42]. Globally and even in high-income countries, an inadequate maternal micronutrient status is common with the evolution of food practices [41,43]. Studies in pregnant women in Germany and secundigravid women in Ireland showed that specific intakes of iron, folate vitamin D, vitamin B_12_ and iodine were below national guidelines, thus highlighting that multiple micronutrient deficiencies can still be prevalent in developed countries and targeted supplementation may be required in certain groups [44,45]. For example, foetal hypothyroidism (most commonly caused by iodine deficiency) may need to be addressed in the European population where only 8 out of 21 countries showed an adequate iodine status (38%) possibly due to low intake of iodine-rich seafood [46].

Folate is a key micronutrient for early stages of placental development [47] and the importance of periconceptional folate intake for child neurodevelopment has been widely recognised [48]. Folic acid supplementation is recommended in the preconception period (at least 3 months before becoming pregnant) and up to the 12th week of pregnancy, to prevent neural tube defects (NTD) and poor neurocognitive offspring outcomes [49,50,51].

Additionally, certain micronutrients are thought to play a role in epigenetic modification pathways including DNA methylation, thus establishing links between early nutrition programming and long-term health trajectories and disease risk [41,48]. Folate; vitamins B_12_, B_2_, and B_6_; betaine; choline; and methionine implicated in one-carbon metabolism (OCM) have been found to change DNA methylation in foetal tissues and in the placenta [19,48,52]. Finally, it has been shown that multiple-micronutrient supplementation may have beneficial effects on the reduction of low birth weight (LBW) and small for gestational age (SGA), [21,22]. Thus, transitioning towards multiple-micronutrient supplementation with evaluation of dietary indices is key in improving placental and neurodevelopment offspring outcomes [21,22,41,53].

### 3.3. Indices of Dietary Quality and Dietary Inflammation

An overview of dietary indices commonly used in the general population is presented in Table 1. The development and construction of these scores in the general population and in pregnant women are discussed further below.

#### 3.3.1. Dietary Inflammatory Index (DII)

First validated in 2009 (Generation 1) [31] and later refined in 2014 (Generation 2, a version that includes an improved scoring system and an updated, more complete literature search) by Shivappa et al. [29], the DII combines a range of macronutrients and micronutrients with various non-nutrient naturally occurring chemicals (e.g., caffeine, flavanols) and herbs and spices (such as onion, turmeric, saffron, thyme, rosemary and green and black tea) [29]. The score represents the overall inflammatory potential of an individual’s diet based on 45 pro- and anti-inflammatory food parameters that either increased or decreased circulating biomarkers of inflammation (CRP, IL1β, IL4, IL6, IL10 and TNFα). The index is calculated using a scoring algorithm; a higher score indicates a pro-inflammatory diet, and a lower score indicates an anti-inflammatory diet. Usually, the DII is expressed as an energy-adjusted DII (E-DII) score because relations between energy and nutrient consumption (a key aspect in determining overall inflammatory potential of the diet) varies across age and according to total body mass [30]. It can be derived from any dietary assessment tool (e.g., food records, national surveys) and is universally applicable across all human studies with adequate dietary assessment [30]. It is important to identify and interpret scoring ranges of the DII used in studies because when calculated from all the 45 food parameters, DII scores can range from −8.87 to +7.98. To incorporate consistency in its interpretation, DII scores derived from 25–30 food parameters would lead to a more effective range of −5.5 to +5.5 [30]. Regardless, it is known that the effective range should rarely exceed 11 [30]. In this review, we focus on offspring associations with the refined (Generation 2) DII.

#### 3.3.2. Mediterranean Diet (MD)

Several dietary metrics based on the MD have been created including the Mediterranean Diet Scale (MDScale) developed by Trichopoulou et al., Mediterranean Food Pattern (MFP), the MD Score (MDS), Mediterranean Lifestyle (MEDLIFE) index and the MedDiet score [54,55]. The Mediterranean dietary metric is based on epidemiologic findings of beneficial dietary effects on cardiovascular health, often localised to healthy eating guidelines [63]. The traditional main components are vegetables, legumes, fruits and nuts, unrefined cereals, olive oil, a moderate intake of fish, low to moderate intake of dairy products, a low intake of meat and poultry and moderate alcohol intake during meals. Differences noted between the various MD scores include number of components (nutrients, foods or food groups intake); classification categories for each item; measurement scales; statistical parameters (mean, median or tertiles of daily intake) and the contribution of each component (positive or negative) to the total score.

#### 3.3.3. Healthy Eating Index (HEI)

Originally developed in 1995, the HEI evaluated adherence to key dietary recommendations from the Dietary Guidelines for Americans [64]. The original HEI was used to measure the overall dietary quality, but since then, the HEI has been modified regularly to reflect changes in dietary recommendations [65]. From 2005, the United States Department of Agriculture (USDA) versions of the HEI were based on an energy density approach [43,66]. The HEI-2010 score includes 12 components: 9 assess dietary adequacy (foods that people should consume more of) and 3 assess moderation (foods that people should consume less of) [56]. The HEI-2015 is the most recent iteration based on conformance with the 2015–2020 Dietary Guidelines for Americans [57]. The HEI-2015 uses a scoring system to evaluate food groups. Scores above 80 indicate a “good” diet, while scores below 51 indicate a “poor” diet [58]. An HEI score between 51 and 80 is considered as “needing dietary improvement”. The HEI-2010 and 2015 components are similar with the following exceptions in the HEI-2015: Saturated fat and added sugars replace empty calories and legumes are allocated to the two vegetable and the two protein foods components (thus highlighting the importance of a varied, balanced diet). Considering the overall similarities of the HEI-2010 and 2015 scores, similar diet–disease associations may be expected with both scores.

#### 3.3.4. Alternative Healthy Eating Index (AHEI)

Compared to the HEI, the AHEI-2010 incorporates foods, nutrients, dietary recommendations predictive of chronic disease risk (i.e., T2DM) and mortality [58]. It consists of 11 components of varying proportions: Six components for which higher intakes are better (vegetables, fruit, wholegrains, nuts and legumes, long chain omega-3 fatty acids (FA) that include docosahexaenoic acid (DHA) and eicosapentaenoic acid (EPA) and polyunsaturated fatty acids (PUFA)); one component for which moderate intake is better (alcohol); and four components that must be limited or avoided (sugar sweetened drinks and fruit juice, red and processed meat, trans fats and sodium) [58]. Hence, the AHEI emphasises diet (predominantly fat) quality (i.e., intakes of omega-3 FAs and PUFAs) and promotes the intake of nuts and legumes using a food-based dietary guideline approach. This could be important in reducing adverse outcomes for infants for women who are at greater risk of gestational diabetes and hypertensive disorders of pregnancy [58,66]. In addition, the AHEI-2010 recommends limiting the intake of red and processed meats and to avoid added sugars (i.e., sugar-sweetened beverages and fruit juice), both of which have been associated with pro-inflammatory states and the risk of overnutrition coming from excess prenatal sugar intakes [67,68].

#### 3.3.5. Dietary Approaches to Stop Hypertension (DASH)

The DASH score was developed for the therapeutic reduction in blood pressure based on dietary patterns and food/nutrient components from the DASH Trial [61]. It is promoted as a healthy option for the general population as a therapeutic means of controlling high blood pressure without the use of medication. The DASH score created by Fung et al. [60] is the most commonly used dietary score in the literature and is relevant to the pregnant population as it was calculated based on a middle-aged female population in the US (a Western developed, industrialised nation) [60,69]. It uses scoring based on the quintile distribution of intake on the following eight components: A high consumption of fruits, vegetables, nuts and legumes; low-fat dairy products and whole grains; and a low consumption of sodium, sweetened beverages and red and processed meats [26]. A DASH score between 8 to 40 (indicative of lowest and highest adherence respectively) ranks participants according to their dietary intakes.

#### 3.3.6. Modified Dietary Scores for Pregnancy

Many of the aforementioned dietary scores have been modified and adapted for characterising maternal diet quality during pregnancy. Mediterranean diet scores such as Trichopolou’s MDS and Khoury’s score have often been modified in observational studies for pregnancy to provide the most useful information in terms of conceptual suitability and applicability [55]. For example, the original a priori Trichopolou’s MDS and the KIDMED index (another MD score validated in younger individuals [2–24 years]) have been modified to consider dairy to be protective due to increased calcium requirements during pregnancy and hence is scored positively [70,71,72], whereas nuts can sometimes be excluded from dietary scores (Khoury and AHEI for pregnancy) as some women may avoid nuts during pregnancy out of concern for allergen sensitisation [73,74,75]. The HEI-SGP (generated based on dietary guidelines and recommendations for pregnant women in Singapore) and the Alternate Healthy Eating Index for Pregnancy (AHEI-P) are conceptually similar to the standard HEI except they include adherence to antenatal supplementation of key micronutrients (iron, folate and calcium) in their scoring [20,75,76]. These scores have been shown to be useful in identifying women ‘at risk’ of poor blood glucose control and pre-eclampsia during pregnancy, who would benefit from early intervention with multiple-micronutrient supplementation [75,76]. The DASH dietary pattern, modified in line with trends observed in the pregnant population such as limiting sugar-sweetened beverage intake, has also been shown to improve glucose tolerance and lipid profiles of women with GDM [77,78]. In the ROLO study, a DASH dietary pattern during pregnancy was associated with lower maternal blood pressure among healthy women without hypertensive disorders of pregnancy [79].

In summary, these specific dietary metrics all share certain characteristics such as a diet high in vegetables, legumes and fruits, indicative of better diet quality and sustained habits, and were associated with one or more important maternal health outcomes such as pre-eclampsia, GDM, altered blood glucose metabolism and postpartum obesity [70,75,79,80,81,82,83]. Additionally, most of these pregnancy-specific dietary tools account for adequacy of key food groups and micronutrients that favour maternal and child health and can serve as useful tools to indicate adherence to national healthy eating recommendations [20,75,76,84].

## 4. Maternal Dietary Metrics and Offspring Birth Outcomes

As mentioned above, studies investigating maternal diet have mainly focused on adequate intake of selected macronutrients and micronutrients during pregnancy. In recent years, there has been growing interest in examining associations between overall maternal diet quality (by using the previously described scores) and child development. Here we summarise the literature regarding the relationship between maternal dietary quality and dietary inflammatory potential with birth outcomes related to growth and prematurity.

### 4.1. Dietary Inflammatory Index

Chronic inflammation during pregnancy that is observed in obese women may alter physiological mechanisms of the developing foetus. Obesity-induced inflammation is characterised by abnormal cytokine and adipokine levels [85]. Some studies have shown that a higher DII or E-DII score indicating a more anti-inflammatory diet may ameliorate the pro-inflammatory state of pregnancy, particularly in obese women [14,15]. A detailed description of the studies outlined below is reported in Table 2. The Healthy Start Study (*n* = 1078 mother–neonate pairs) reported that higher DII scores in obese mothers during pregnancy were associated with increased neonatal adiposity [86], while the NEST study (*n* = 1057 mother–child pairs) did not report associations with birth anthropometry but an increased risk of prematurity among female offspring was observed [14]. Conversely, in the Lifeways study, higher maternal E-DII scores in early pregnancy were associated with increased risk of low birthweight [24]. Additionally, results from the Project VIVA showed that higher DII scores were associated with lower birth weight for gestational age *z* score in infants born to obese mothers [15]. There are limited investigations of maternal DII and its influence on birth outcomes, and results remain inconsistent. However, a recent meta-analysis of individual participant data on 24,861 mother–child pairs, in seven European cohorts, has been recently conducted [87]. A higher maternal pre-pregnancy E-DII score was associated with lower birth weight [β(95% CI) = −18.7(−34.8, −2.6) g per 1-SD higher E-DII score] and shorter birth length [−0.07(−0.14, −0.01) cm], whereas a higher E-DII score during pregnancy was associated with an increased risk of LBW [odds ratio (OR)(95% CI) = 1.14(1.04, 1.26) and being small for gestational age (SGA) [OR(95% CI) = 1.18(1.11, 1.26)] and shorter birth length [−0.06(−0.10, −0.01) cm. These findings, which were robust across a range of sensitivity analyses, highlight the importance of having a less pro-inflammatory diet not only during pregnancy but also before becoming pregnant.

### 4.2. Mediterranean Diet

Some studies have examined the potential benefits of the Mediterranean diet on foetal development, considering the MD as a whole rather than focusing on the effect of its individual components. Foetal growth restriction (FGR) and SGA were the main outcomes examined. In a prospective observational study of 3207 Caucasian pregnant mothers in Rotterdam, low adherence to a MD in early pregnancy was associated with decreased intra-uterine size with a lower placental and a lower birth weight [88]. Inversely, in the INMA-Mediterranean cohort in Spain, mothers with high MD adherence had a significantly lower risk of having foetal growth restricted infant at birth [89]. However, the previous results were not consistent in the INMA study from the Atlantic area and the RHEA cohort in Greece [89]. One limitation of these studies is that the MD is subject to some regional variations associated with sociocultural, population and geographic differences. MD scores during pregnancy were not homogeneous, with women in the Atlantic area reporting higher intakes of fish and dairy products, while women in Greece reported higher intakes of fruits and nuts. These observations could explain in part the different results in population. However, in all cohorts, they showed a protective effect of adherence to MD in smokers that may counter the effect of oxidative stress damage on foetal tissues [89].

Other studies reported associations between maternal MD and prematurity. In a study from the French West Indies, where dietary patterns present several characteristics of the MD diet, no overall associations were found between MD adherence and birth weight. However, there was a decreased risk of prematurity associated with greater MD in overweight and obese women [90]. In the observational cohort of 35,530 Danish women, Mikkelsen et al. showed a 72% reduction in the risk of early preterm delivery (<35 weeks of gestation) in women who fulfilled all MD criteria, compared to women fulfilling none of the MD criteria during pregnancy [91]. Meanwhile, another study in Norway, which defined MD with the same criteria as the previous study, showed no association with risk of prematurity [73]. However, several hypotheses explaining the potential link with reduced risk of prematurity have been described. In particular, one randomized trial has evaluated the effect of a cholesterol-lowering diet on maternal pregnancy outcomes. In that study, women in the intervention group were advised to change their diet towards a Mediterranean-type diet. A diet enriched with n-3 (omega-3) PUFA may influence the level of inflammatory cytokines, which are involved in the pathophysiological mechanisms of prematurity and reduced its incidence [74]. Most studies focused on evaluating birth weight or prematurity; however, a recent study showed that greater MD adherence was positively associated with birthweight, length and sum of skinfold thickness, a measure that better captures total neonatal adiposity [92]. A detailed description of the previous articles (non-exhaustive list) is reported in Table 3.

To conclude, two recent systematic reviews of literature reported growing evidence of the potential benefits of the MD diet for child development, but other randomized trials are needed to confirm these results [80,98]. Moreover, the strength of the association varied in the different studies, which can be affected by high heterogeneity related to the methodology used for the MD assessment. The number of food items included in the FFQ, the way of administration and the calculation of consumed foods may explain some differences between results. Another confounding factor is the period of pregnancy considered for evaluation of MD adherence [98]. 

### 4.3. HEI, AHEI and AHEI-P Scores

Several studies have examined birth outcome associations with these dietary indices (see Table 4). In a Spanish cohort, Rodriguez-Bernal et al. found that increasing quintiles of the AHEI-P score were associated with higher birth weight and length [99]. However, the results remain inconsistent, with several studies reporting no association between maternal AHEI-P or AHEI-2010 scores and anthropometry measurements at birth [75,100,101]. Other studies reported that low maternal HEI-2010 scores were associated with offspring adiposity and risk of being large for gestational age (LGA) [102,103]. Most of the studies of maternal diet quality during pregnancy used previous versions of HEI scores, while another study reported a lower risk of LBW with a high adherence to the HEI-2015 [104]. Variations in the dietary assessment, as some of them were updated for pregnancy and participant characteristics, are the main contribution to the discordance in the results. However, higher adherence to these indices have been strongly linked to a greater dietary variety and consumption of essential nutrients that may lead to a favourable cardiometabolic profile in pregnant women [103].

### 4.4. DASH

A systematic review and meta-analysis of RCTs provides evidence that the DASH diet could play a role in glycaemic control and improve gestational outcomes in women with cardiometabolic disorders such as GDM, hypertension or obesity [107]. Among the studies included, one revealed that adherence to the DASH diet for 4 weeks among pregnant women with GDM was associated with improved fasting glucose profile and reduce the use of insulin therapy. Mean birthweight, head circumference and ponderal index of infants born in the intervention group were significantly lower compared with those born to mothers in the control arm [78]. Other observational and RCTs report that adherence to the DASH diet in women with risk factors (obesity, hypertension) was associated with lower birthweight or birth length, a lower risk of prematurity or having a LGA infant [82,108]. Inversely, another RCT conducted in a group of overweight and obese women failed to demonstrate improved birth outcomes with a DASH diet, even when a reduction of gestational weight gain was observed [109]. A description of the few studies included is shown in Table 5. The published literature on the relationship of the DASH diet with birth outcomes remains inconsistent. Differences between studies regarding the diagnosis of GDM, hypertension or obesity might explain fundamental physiological differences between the included women [110]. Future studies including more generalisable populations are needed to ascertain the effect of the DASH diet [107]. A recent meta-analysis of the ALPHABET European project, conducted in a general population of pregnant women, has shown that a higher pregnancy DASH score was associated with higher birth weight [β (95% CI) = 18.5 (5.7, 31.3) g per 1-SD higher DASH score] and head circumference [0.03 (0.01, 0.06) cm], as well as longer birth length [0.05(0.01, 0.10) cm], and lower risk of delivering SGA [0.87(0.82, 0.94)] infants [87].

### 4.5. GI/GL

A recent meta-analysis of 11 randomized trials including 1985 women has shown that compared with control diets, a low GI diet significantly reduced fasting and 2-h postprandial blood glucose levels and proportion of LGA infants [36]. However, some of the RCTs were conducted mostly in high-risk populations (with a history of gestational diabetes or previous macrosomic infant) and differed in terms of study design, which may contribute to heterogeneity among the studies and may partly explain the inconsistent results [81,112] (Table 6). The ROLO study is an RCT of 800 secundigravida women who had previously given birth to a macrosomic baby (>4 kg), randomised to low GI dietary advice vs. usual antenatal care, which did not involve dietary advice [81]. No differences between groups were reported in birth weight or 6-month offspring adiposity [81,113], whereas another RCT conducted in women with diabetes showed a trend of lower birth weight after a low GI carbohydrate intervention compared to all types of carbohydrates [112]. Other observational studies reported controversial results between GI/GL and birth outcomes [35,38,40,83]. A prospective cohort in Ireland evaluated the associations between maternal GI, GL, insulinemic index/load (II, IL) and offspring birth outcomes. Dietary II and IL may play an important role in blood glucose regulation [38]. No associations between glycaemic or insulinemic indices during early pregnancy with birth weight, BMI at birth or gestational age were observed [38]. In summary, some studies indicate reduced risk of adverse neonatal outcomes following a low-GI dietary intervention. However, the evidence is limited, and results should be interpreted with caution because of the evidence of heterogeneity across studies.

## 5. Maternal Dietary Metrics and Offspring Childhood Outcomes

In line with the DOHaD hypothesis, maternal diet associations with offspring health may not be restricted to birth and may have longer term implications. Here we summarise the literature regarding the relationship between maternal dietary quality metrics and dietary inflammatory potential with offspring outcomes related to childhood growth and obesity. A summary of the studies and their key findings is presented in Table 7.

### 5.1. DII

Data regarding long-term associations of maternal dietary inflammation with offspring anthropometric outcomes are scarce. Sen et al. reported positive associations between maternal DII (measured during the first and second trimesters) and offspring anthropometric measures in mid-childhood (between ages 6 and 10), which included BMI z-score and waist circumference as well as more direct measures of adiposity such as fat mass index, fat-free mass index (FFMI), trunk fat mass index and fasting insulin [84]. The E-DII score was also recently analysed in the ALPHABET Consortium in 16,295 mother–child pairs from seven European birth cohorts. Higher early-pregnancy E-DII scores (indicating a more pro-inflammatory diet) tended to be associated with a higher odds of late-childhood [10.6 (1.2) years] overweight and obesity (OWOB) [OR (95% CI) 1.09 (1.00, 1.19) per 1-SD E-DII score increase], whereas an inverse association was observed for late-pregnancy E-DII score and early-childhood [2.8 (0.3) years] OWOB [0.91 (0.83, 1.00)]. In two cohorts with available data, a higher whole-pregnancy E-DII score was associated with a lower fat-free mass index in both mid-childhood and late-childhood [123]. In the Irish Lifeways cohort, there were no associations found between maternal dietary inflammation during pregnancy and childhood adiposity, whereas paternal line dietary inflammation appears to influence childhood obesity at both 5 and 9 years [24].

### 5.2. Mediterranean Diet Score

Chatzi et al. studied anthropometric outcomes in 997 mother–child pairs in the American Project Viva study in conjunction with 569 mother–child pairs from the Greek Rhea study. They found that maternal adherence to the MD during pregnancy was associated with lower child adiposity, leptin and blood pressure levels [120]. It is important to note that the Greek cohort is based on outcomes in early childhood at around 4 years compared to the American median age of 8 years. However, they report that these associations of MD scores in pregnancy with offspring adiposity were broadly similar in both cohorts when studied separately [89,120].

The analysis of results from the Growing Up Today Study II across follow-up ages of 12–23 years indicate that greater maternal adherence to the Alternate Mediterranean Diet (aMED) score (adapted to the US population) showed similar findings with a lower risk of offspring overweight or obesity [116]. However, the results were not significant following adjustment for potential confounders including maternal pre-pregnancy BMI and lifestyle factors. It should be noted that childhood weight measures were self-reported in the study, which may lead to underreporting amongst adolescents with obesity [116]. In a Spanish study predicting long-term health outcome risks, the importance of tracking childhood adiposity rather than crude weight alone is underscored, because cardiometabolic risk factors are more prevalent in children and adolescents with abdominal obesity than those with general overweight/obesity [121,124,125]. The researchers reported that higher adherence to the relative Mediterranean diet score (rMED) in pregnancy was not associated with offspring overweight status at age 4 years. However, there was some evidence of an inverse, moderate association between the rMED in pregnancy and offspring waist circumference, a marker of abdominal obesity [121]. Furthermore, Donahue et al. have demonstrated a protective effect of prenatal omega-3 PUFAs (which is a key macronutrient in the Mediterranean diet) on adiposity in 3-year-olds [126].

### 5.3. HEI and AHEI Scores

Tahir et al. report that higher HEI-2015 scores during the third trimester of pregnancy were associated with lower infant body fat percentage at six months [119]. However, another study by Gonzalez-Nahm et al. in an American cohort found no association between AHEI-2010 scores in mid to late pregnancy and infant adiposity at birth, 6 or 12 months after adjustment for confounders such as maternal smoking, pre-pregnancy BMI, maternal ethnicity and maternal education [118]. Similarly, retrospective analysis by Strohmaier et al. found no associations between peripregnancy maternal adherence to the AHEI-2010 and offspring risk for obesity 12–23 years after adjustment for pre-pregnancy BMI and lifestyle factors [116]. Long-term studies using the modified AHEI-P are not yet available.

### 5.4. DASH Score

Despite its importance in controlling blood pressure and placental vascular flow, research regarding maternal DASH score associations with offspring adiposity and childhood obesity are scarce. Strohmaier et al. report an inverse association between peripregnancy DASH scores and offspring overweight risk at 12–23 years; however, this association was no longer observed after adjusting for maternal BMI and lifestyle factors before pregnancy [116]. Recent results from the ALPHABET Consortium in 16,295 mother–child pairs from seven European birth cohorts showed that higher maternal DASH score during pregnancy was associated with a lower odds of late-childhood OWOB (age-and-sex-specific BMI *z*-score >85th percentile) as well as a lower late-childhood fat mass index (FMI) [123]. Interestingly, no consistent associations were observed between maternal DASH scores with regards to *early*- and *mid*-childhood OWOB (mean age range 2.8–6.1 years) [123].

### 5.5. GI/GL

Results from the Southampton Women’s Survey of 907 mother–child dyads identified associations between higher maternal GI and GL with greater offspring adiposity (body-fat mass) at 4 and 6 years [122]. These results were found with early, but not late pregnancy GI and GL, highlighting the importance of the timing of the dietary assessment during pregnancy in the programming of adult disease susceptibility. However, more recent observational research based on the Lifeways cohort of generally healthy pregnant women found no clear associations between maternal GI and GL with childhood BMI, waist circumference z-scores and general or central obesity at 5 years [38]. In the Danish National Birth Cohort, higher maternal GI and GL were associated with an increase in 7-year BMI *z*-score in the unadjusted analysis. However, this association was no longer present after adjusting for previously mentioned confounders, particularly maternal pre-pregnancy BMI [117]. Similarly, a secondary adjusted analysis of the Irish ROLO cohort found no evidence that a low-GI diet in pregnancy impacts offspring anthropometry and body composition outcomes after 5 years’ post-intervention [115]. Limited and inconsistent literature exist regarding maternal dietary scores and long-term childhood outcomes related to weight status and adiposity.

## 6. The Role of the Placenta during Pregnancy

### 6.1. Placental Development

The placenta plays a fundamental role in the various maternal-foetal exchanges during pregnancy and has endocrine functions that are essential for the maintenance of the pregnancy as well as for the development of the foetus [16]. Together with the umbilical cord, it provides for the direct transfer of nutrients, hormones and wastes between the mother and offspring. Originally formed from trophoblast cells during the implantation phase, the growth rate of the placenta is most rapid in the first trimester of pregnancy, a time when placental tissues are most sensitive to environmental perturbation [127,128]. Inadequate maternal nutrition or weight status can have major effects on the balance between the nutritional needs of the foetus and the placental capacity to meet them [41,129].

Different markers of altered placental function reflected by weight, morphology, vascular development and transport function have been described. Markers of modifications in placental transport capacity include alterations of exchange surface area, barrier thickness and cell components of the different gestational ages of the placenta. These histopathological changes predispose to reduced nutrient transfer to the foetus. Additionally, vasculogenesis and angiogenesis are both critical processes to the maternal-foetal exchange. Vasculogenesis is de novo formation of blood vessels in the developing embryo while angiogenesis corresponds to new vessels from pre-existing vessels [17]. The foetal (birth weight) to placenta weight ratio at different gestational ages of placental development also been used, but collecting these measurements is often difficult in human studies [17,130]. Therefore, placental weight has been described as the most practical, studied marker of the available surface area for maternal foetal nutrient exchange [130,131,132]. Despite not being the only marker available, investigating the effects of dietary quality on placental weight could provide important insights, which may inform dietary guidelines or interventions to help prevent abnormalities in placental and subsequent child development.

Alterations in maternal nutritional status, such as undernutrition, have been described as impairing placental development and function, including placental weight, its function in transporting various nutrients, as well as its vascular development (de novo vessel formation) and the process of angiogenesis [17]. The pathogenesis of blood flow dysfunction such as pre-eclampsia may be established in early pregnancy when an inadequate remodelling of the spiral arteries occurs and leads to decreased perfusion of the placenta and development of the maternal syndrome in later pregnancy [133]. Oxidative stress and inflammatory mediators are also involved in the abnormal implantation associated with pre-eclampsia and IUGR, thus highlighting the role of diet quality in optimal implantation and placentation during the preconceptional period [41,133]. Consequently, inadequate placental development could lead to inappropriate foetal development and risk of IUGR [41,129,133]. Assessing the underlying vascular and inflammatory mechanisms associated with placental perfusion and transport efficiency is difficult in humans and most research has been limited to experimental animal models, specifically rats, mice and sheep. Conversely, other studies showed that maternal nutritional conditions such as hyperglycaemia and hyperinsulinemia are also likely to contribute to alterations in placental growth and weight [134]. However, more research is needed in investigating how early nutritional and metabolic change during pregnancy may affect placental development and lead to excessive neonatal adiposity. It is also important to note sex-specific placental adaptations that may drive differences in offspring growth [135]. In females, increased foeto-placental adaptability and placental reserve capacity result in increased survival rates at the expense of a reduced growth trajectory, whereas the opposite is observed in males [135]. Recent studies highlighted that sexual dimorphism may play a role in the impact of the placenta on infant anthropometry at birth, with significant findings seen in males but not females [136].

### 6.2. Maternal Diet and Placental Development: Evidence from Animal Studies

Starting with animal studies, some researchers have looked at the associations between high-fat diet or micronutrient and placental development [137]. Feeding pregnant rats a ‘junk’ food diet, characterised by high fat, high sugar and high salt, has demonstrated increased risk of adiposity in adult offspring [138]. These energy-dense diets often lead to overnutrition that is thought to elicit metabolic disturbances in placental development as well as foetal hyperglycaemia and hyperinsulinemia [127,128]. Across periconceptional rodent studies, there were no differences in foetal and placental weights where high-fat diets were given to rats compared to the control [139,140]. However, altered placental gene expression, with pathways promoting inflammation and growth being over-represented among up-regulated genes and those affecting apoptosis among down-regulated genes, has been observed following maternal high-fat feeding in pregnant rats [139]. Thus, multiple rodent studies highlight the impact of a high-fat diet on placental metabolism and thus on foetal programming for long-term offspring adiposity [141,142].

Other studies also evaluated the effect of specific micronutrients on placental development and inflammation. Reduced placental weight has been reported in severe iron deficiency in rats [137]. This is of note as further rat studies show that anaemia induces oxidative stress and inflammation in rat placentas, with elevated circulating levels of pro-inflammatory IL-6 and TNF-α as well as oxidative damage (lipid peroxidation) and reduced antioxidant status [137,143]. Folate is directly able to scavenge free radicals while B vitamins (B_2_, B_6_ and B_12_) also have antioxidant activity, thus playing an important role in early stages of placental development, including trophoblast invasion and angiogenesis [47,133]. Moreover, folate-deficient mice were found to have decreased foetal weight and foetal/placental weight ratio [144]. Folate in particular is thought to play a key role in placental angiogenesis (critical for development of a normal placental circulation) by increasing the bioavailability of nitric oxide [17,47]. This is an important endothelium vasodilator, and its decreased bioavailability is associated with the production of reactive oxygen species involved in poor placental development [133,145]. Furthermore, maternal vitamin B_12_ deficiency in rats altered placental phospholipid ratios, underscoring the potential of micronutrients in influencing placental development and composition [137].

### 6.3. Maternal Diet and Placental Development: Evidence from Human Studies

Maternal nutritional status during pregnancy plays a major role in foetal development, and accumulating evidence suggests that maternal hyperglycaemia may have adverse effects on embryonic and placental development [35]. Observational studies in the UK and Ireland have found that high carbohydrate intake during pregnancy could impair maternal insulin sensitivity, making higher levels of free glucose available for placental circulation, subsequently activating foetal glycogenesis and contributing to excess neonatal adiposity [21,67,146]. Such maternal overnutrition and as well as diabetes can also cause transient physiologic proinflammatory oxidative stress in the trophoblast, leading to a reduction in trophoblast growth in the placenta [128,134]. The link between dietary fat composition (fatty acid quantity and quality) and chronic disease risk is well established [147,148]. Maternal dietary fat intake has been shown to affect the fatty acid composition of lipids in foetal and newborn blood and developing tissues. By affecting the fatty acid supply to the foetus, differentiation in organs including the placenta can occur, which may in turn influence placental development and foetal programming [127,148].

Excessive omega-6 fatty acid intake and insufficient omega-3 fatty acid intake are two key components that adversely affect foetal programming [148]. An Icelandic observational study reported an inverse association between the proportion of long-chain omega-3 PUFAs in red blood cells of women in the 11th to 15th week of pregnancy and placental weight [133]. A moderate maternal dietary protein intake (10–25% of total energy) is optimal for foetal growth and survival, as both low but also high protein intakes have been associated with lower birth weights and IUGR [21,149]. Possible mechanisms include reduced placental angiogenesis and reduced supply of nutrients from mother to foetus for low protein intakes. High maternal concentrations of amino acids (AA) leads to competition for AA transporters, resulting in reduced placental transport and umbilical uptake of AAs [149].

Other studies have evaluated the effect of micronutrients on placental development. In a prospective study of 1650 pregnancies, low iron status in early pregnancy was inversely related to placental size [150]. The synergistic use of vitamin E and vitamin C as antioxidants are important to consider in placental development due to their effect of inhibiting free radical formation to prevent oxidative stress and inflammatory placental damage [21]. Studies have demonstrated reduced placental perfusion and risk of pre-eclampsia arising from the placenta’s limited antioxidant enzyme capacity in the first trimester [133]. The micronutrient immunomodulatory effects of Vitamin A, Vitamin D, folate and B_12_ in in vitro/in vivo models of deficiency have also all demonstrated pro-inflammatory effects in the placenta including upregulation of pro-inflammatory cytokines [137].

However, research on maternal dietary score associations with placental development is scarce. In the Generation R cohort, women with low adherence to a Mediterranean diet pattern during early pregnancy had smaller placentas and also tended towards higher uteroplacental vascular resistance compared to those with high adherence to the MD pattern. However, it is important to note that an established, validated MD score was not used in this study [88]. Recent observational analysis in the same Dutch cohort found that in mid- and late- pregnancy, a higher maternal DASH diet score based on Fung’s scoring method tended to be associated with lower umbilical artery vascular resistance and that lower maternal DASH score quartiles were associated with a higher umbilical artery pulsatility index (UmPI) [151]. The UmPI reflects the development of the fetoplacental vascular tree. Studies using the DII are more limited but early research suggests that oxidative stress or inflammation caused by a pro-inflammatory diet results in higher resistance and lower flow placental circulation [15]. This resultant mechanism leading to adverse foetal growth is thought to be more pronounced in obese pregnant women, who experience higher baseline inflammation and oxidative stress [15]. Further research on all of these validated maternal dietary score associations with placental development and inflammation is warranted.

## 7. Epigenetic Mechanisms

In 1942, Conrad H Waddington defined epigenetics as “the branch of biology that studies the causal interactions between genes and their products which bring the phenotype into being” [152]. Epigenetics was subsequently defined as the study of the mechanisms that regulate gene expression without alteration in the DNA sequence [153]. Epigenetic processes are essential for many organisms functions, but can be associated with major adverse health effects if they occur inappropriately [154]. Three main mechanisms are described in the literature: DNA methylation, histone modifications and noncoding RNAs [155]. DNA methylation, which is currently the easiest marker to measure and therefore the most studied in epidemiological studies, is the focus of this review.

### 7.1. DNA Methylation and Developmental Plasticity

Epigenetic modifications have been proposed as mechanisms by which a suboptimal nutrition state could contribute to the “developmental plasticity of the individual”. Early-life exposures have been described to be recorded in the “cellular memory” and having the potential to influence cellular functions with long-term effects [18,156]. The periconceptional period is a particular sensitive period for environmental exposures, due to major epigenetic processes that take place at the beginning of pregnancy [18]. Upon fertilisation, a global demethylation is observed, followed by de novo genome-wide methylation after implantation [18]. Thereafter, new methylation patterns are established from pluripotent cells in a lineage-specific manner leading to organ and tissue differentiation [18,157]. However, other studies showed that epigenetic marks are dynamic and modifiable under environmental conditions throughout the pregnancy and development [158] Several discoveries have highlighted the importance of DNA methylation for embryonic development, cell differentiation during development and the maintenance of tissue-specific gene expression patterns [18,159]. An inadequate environment during the periconception period may have an impact on epigenetic mechanisms, foetal programming and future health. Thus, the in utero environment, in particular maternal nutrition, has been described as one of the important factors in the modulation of gene expression [157]. This chapter reviews the current state of knowledge of maternal nutrition in DNA methylation changes. This review includes studies related to over- or undernutrition, glycaemic markers, the one-carbon metabolism (OCM) nutrients and a focus specifically on dietary quality and inflammation.

### 7.2. Maternal Nutritional Intake and DNA Methylation

The first evidence is based on findings from animal studies. Murine models have provided evidence about how early nutritional exposure is involved in epigenetic mechanisms, programming of the individual and its phenotype [18]. A classic example is the agouti gene, which is involved in determining coat colour in mice. In genetically identical mice, some have a brown coat and others a yellow coat [18]. The agouti viable yellow mouse is characterised by a yellow coat colour and an increased risk of obesity and hyperinsulinaemia. This is caused by a dominant mutation of the agouti locus, caused by the insertion of a so-called IAP (intracisternal A particle) repeat element, which acts as an alternative promoter. Hypomethylation of the promoter led to overexpression of the gene involved in the regulation of yellow pigment and other effects including obesity [18]. Conversely, when the percentage of methylation increases, the synthesis of the yellow pigment is regulated downwards, resulting in a brown or “pseudoagouti” coat [18]. One study showed that methyl-donor supplementation (folic acid, vitamin B_12_, choline and betaine) was associated with hypermethylation of the agouti gene [160].

Recent human data are becoming available regarding the associations between maternal diet and DNA methylation. It has been described that some micronutrients are implicated in the one-carbon metabolism pathway, which supports multiple physiological processes, essential for human development including DNA methylation [19,161]. The main studies have focused on the associations between individual micronutrients, methyl-donors and DNA methylation and the role of maternal over- and undernutrition. However, there are a limited number of human studies that have evaluated the relationship between dietary quality or inflammation state and offspring epigenetics.

#### 7.2.1. One-Carbon Metabolism Nutrients

The Pune Maternal Nutrition Study cohort in India have provided evidence of the importance of the one-carbon metabolism in foetal programming [53]. OCM nutrients are described as carriers or methyl-group donors (e.g., folates, choline, betaine, methionine), or cofactors of enzymes involved in the transfer reactions of the methyl-groups to DNA (vitamins B_2_, B_6_, and B_12_) [19]. Some studies have shown the implication of these micronutrients in offspring DNA methylation. Maternal dietary and supplemental intake of methyl-groups donors (folate, folic acid, betaine) in the preconception period can influence an infant’s buccal DNA methylation patterns at 6 months old. The identified genes were associated with development, metabolism and appetite control [162]. In Gambia, it has been found that significant seasonal variations in maternal methyl-donor nutrient intake around the time of conception may influence levels of DNA methylation at metastable epialleles in offspring [163,164]. Metastable epialleles are genomic regions whose expression is determined in the early embryo and independently of cell differentiation [165]. Modifications at metastable epialleles can lead to permanent phenotypic consequences [163,165]. Another study has investigated the relationship between serum levels of folate, vitamin B_12_ and methylation of the imprinted genes insulin-like growth factor 2 (*IGF2*) in maternal and cord blood. *IGF2* is a key factor in human growth and development. The methylation patterns of *IGF2* in promoter 3 of cord blood samples were negatively associated with serum levels of vitamin B_12_ only in maternal blood [166]. Other results reported significant associations between maternal folic acid intake in early pregnancy and offspring DNA methylation in the control of imprinted genes (*IGF2*) and transposable elements such as long interspersed nuclear element 1 (*LINE-1*) [18,167]. One RCT has confirmed these results by showing that folic acid supplementation during pregnancy compared with placebo results in significant changes in cord blood DNA methylation in genes involved in growth and brain development [168]. However, most of the previous studies are observational and, thus, by design cannot provide direct causal link between micronutrient intake and epigenetic effects in offspring. Moreover, further research is needed investigating the combined intake of OCM nutrients, the interactions among nutrients and those that may indirectly affect the DNA methylation process [19].

#### 7.2.2. Interventional Studies on Glycaemic Index

In recent years, some interventional studies have focused on the impact of GI dietary intervention in mothers during pregnancy and evaluated their relation to DNA methylation in the progeny. Exposure to adverse conditions during pregnancy like GDM is increasing and has been largely shown to affect foetal development [169]. New evidence also supports the epigenetic role in metabolic development of newborns exposed to maternal hyperglycaemia during pregnancy [169]. The ROLO study, an RCT, investigated the impact of a low GI dietary intervention during pregnancy on DNA methylation in cord blood at birth by using a genome-wide approach [170]. No individual probes remained significantly differentially methylated between the intervention and control groups after adjusting for multiple tests. Nevertheless, hierarchical clustering of the top 1000 unadjusted differentially methylated probes revealed two methylation clusters in the intervention study and maternal BMI difference between the two groups (intervention/control) [170]. In the same cohort, they also recently evaluated the epigenetic patterns on saliva samples of 63 infants at 5 years old, following the intervention during pregnancy. Gene pathway analysis identified three functional clusters involved in insulin secretion and resistance for which there were differences between the intervention and control groups [171]. One small clinical trial of 24 women has provided evidence suggesting that maternal dietary GI modifications during pregnancy may affect placental DNA methylation of insulin regulation genes [172]. The UK Pregnancies Better Eating and Activity Trial (UPBEAT) RCT has evaluated the effect of lifestyle intervention (low GI diet plus physical activity) v standard care in 557 pregnant women with obesity. They showed that the methylation changes observed with maternal GDM exposure appeared to be reduced by the pregnancy lifestyle intervention [173]. However, interventional studies on maternal diet during pregnancy as assessed by candidate gene approach remain scarce.

#### 7.2.3. Maternal Under- and Over-Nutrition

Several animal studies have reported that maternal overnutrition or dietary restriction were associated with direct consequences on foetal development. Some investigations conducted in murine models reported that maternal overnutrition programs long-term epigenetic modifications [174,175,176]. A study on maternal high-fat (HF) diet during 4 weeks before and during gestation caused hyperglycaemia and insulinemic resistance in male offspring as well as modification in gene methylation in the offspring liver [174]. In another study, female rats were fed low-fat or HF diet for 6- weeks before pregnancy and during pregnancy. Maternal overnutrition was associated with long-term epigenetic alterations in the offspring’s hypothalamic Proopiomelanocortin (*POMC*) promoter. The *POMC* gene is described as a key factor in the control of energy balance [175]. Other results indicate that modest dietary protein restriction during pregnancy may alter the phenotype through epigenetic changes in specific genes [177].

Epidemiological studies conducted in humans have also linked the impact of maternal under- and over-nutrition during critical periods of development with the child development [178]. One of the most significant studies was conducted in children from mothers exposed to the Dutch famine during the periconceptional period and reported a reduced level of methylation of the imprinted gene *IGF2* in the adulthood [179]. These results reinforce the periconception period as a crucial period for maintaining epigenetic marks. Recently, in the context of increasing prevalence of childhood obesity, several studies have evaluated the impact of maternal obesity or excessive gestational weight gain on many adverse child outcomes through epigenetic mechanisms. In the Newborn Epigenetics study (NEST) cohort, maternal pre-pregnancy BMI was associated with CpG variations measured in cord blood leukocytes [180]. It has been shown that the methylation differences at CpG sites located in the TAP Binding Protein (*TAPBP*) gene were associated with BMI z-score and systolic blood pressure (BP) percentile in female and systolic and diastolic BP percentile in male offspring at 5 years [180]. Recent observations from the Avon Longitudinal Study of Parents and Children (ALSPAC) cohort identified several CpG sites that are differentially methylated in the cord blood of offspring of obese mothers compared with offspring of normal weight mothers [181]. However, no association between gestational weight gain and DNA methylation has been found [181]. A meta-analysis of 19 cohorts found associations between maternal adiposity and variations in newborn blood DNA methylation [182].

#### 7.2.4. Dietary Quality and Inflammation

While more research concerning maternal obesity and individual micronutrient intake and offspring epigenetics has been conducted, far fewer studies have examined dietary patterns or specific types of maternal diets, which yet reflects dietary quality and food interactions.

The MD pattern has also been associated with higher intake of specific nutrients such as folates that have been shown to influence DNA methylation [183]. However, few studies have evaluated the associations between the MD and epigenetic outcomes. The effects of the MD were assessed in 390 mother–child pairs from the NEST cohort, in nine regions of imprinted genes selected for their involvement in embryonic growth regulation and brain development [183]. They observed a decreased level of methylation at the *MEG3-IG* DMR in cord blood leukocyte among girls, in response to lower maternal adherence to a MD pattern in early pregnancy [183]. One other publication from the NEST cohort has shown perinatal MD score associations with sex-dependent methylation differences for imprinted control regions of *MEG3*, *IGF2*, *PLAGL1 and* *SGCE/PEG10* in cord blood samples [184]. Genomic imprinting is an epigenetic mechanism that leads to the monoallelic expression of genes in a parent-of-origin-dependent manner [185]. Imprinted gene DNA methylation is largely established in the developing gametes and maintained in somatic cells, making it as an important indicator of early exposures [183]. There are few published studies on the relations between the MD and epigenetic outcomes; however. the MD has been largely recommended for its ability to reduce inflammation and improve long-term outcomes in adults [183]. Further research is needed to understand the potential mechanisms.

Inflammation has recently been proposed as another pathway by which maternal diet could affect the epigenome [14]. The hormonal and immunological changes that occur during pregnancy are necessary to support a healthy pregnancy and induce an anti-inflammatory state [186]. Chronic inflammation state during pregnancy, observed in obese women, has been associated with many adverse pregnancy outcomes such as prematurity and LBW [14,87]. Emerging evidence showed the role of chronic inflammation during pregnancy on foetal programming, but the underlying mechanisms are still poorly understood [85]. One study tested the association between the E-DII during pregnancy and both offspring methylation of differentially methylated regions of imprinted gene (*n* = 338) and circulating cytokines (*n* = 105) [14]. Although no associations were observed between E-DII and cytokines levels or offspring methylation in infant cord blood, they found that inflammatory cytokine concentrations were associated with lower methylation at the *MEG3* imprinted gene [14]. Despite their null findings between maternal E-DII score and DNA methylation, positive associations were reported with birth outcomes suggesting a potential role of inflammatory diet in child development, irrespective of the underlying mechanism. While limited, there is accumulating evidence of an association between maternal inflammatory conditions and offspring methylation.

In summary, it has been largely shown that specific micronutrients are involved in the DNA methylation process; however, further investigations regarding the potential role of maternal dietary quality or inflammation are necessary.

## 8. Conclusions and Future Directions

### 8.1. Summary and Discussion of Results

In this review, we examined and summarised findings from a growing body of research investigating associations between maternal dietary inflammatory potential and dietary quality, determined by various scores and foetal development, childhood adiposity, placental measures and epigenetics. The main results suggest that diets of the highest quality, as assessed by the HEI, AHEI, MD, DASH and GI/GL scores, during pregnancy are beneficial for a wide range of maternal and offspring outcomes. However, findings in human studies remain inconsistent.

The evidence regarding DII and E-DII scores highlight the importance of having a less pro-inflammatory diet in pregnant women for improving birth outcomes and reducing the risk of prematurity. The findings in relation to dietary quality associations with long-term child development and placenta development are either conflicting or limited. Finally, few studies investigating relationships between dietary scores and DNA methylation exist. Thus, future research investigating the potential influence of dietary inflammatory status and dietary quality on these outcomes is warranted.

The observed heterogeneity of study populations, ethnicity, differences in dietary assessment methods, timing of dietary assessment and outcome measures may contribute to the observed inconsistent results. Most studies are observational in nature, thus causality cannot be established. However, it has been shown that low-GI dietary intervention may lead to decreased risk of adverse neonatal outcomes [36]. These observations encourage future investigation including RCTs. Finally, another limitation related to nutritional epidemiology is the lack of precision in measuring diet in all its complexity. Although some recent scores like the DII were designed to be comparable across populations [30], information on food consumption was self-reported by women in several studies, which might be subject to recall bias and measurement errors.

### 8.2. Future Perspectives

Most of the research to date regarding early life nutritional programming has focused on the mother but emerging evidence suggests that paternal nutritional status may also have an effect on child development, independently of maternal diet [24,104]. In a study conducted in Ireland, higher paternal E-DII scores, indicating a more pro-inflammatory diet, were associated with increased risk of childhood OWOB at 5 years [24], whereas higher paternal HEI scores, indicating higher dietary quality, were associated with reduced risk of childhood obesity [104]. Other studies reported that higher paternal BMI at conception was associated with increased offspring BMI [187]. In an American cohort study including 429 father–child pairs, paternal BMI, independently of maternal BMI, was associated with offspring epigenetic modifications at birth and at ages 3 years and 7 years [188]. Moreover, fathers have been described as important contributors of family food practices [189]. Interventions during the pre-conception period should include both parents and consider other family-based health behaviours such as physical activity and sedentary behaviour, which play an important role. Moreover, recent studies showed sex-specific foetal growth patterns associated with maternal healthy lifestyle [100]. This sexual dimorphism might arise from differences in foetal programming and responses to adverse intrauterine exposure. Further research is needed to understand if these may have different implications on long-term health among males and females [190]. Further research to ascertain mechanisms linking maternal diet with health outcomes in offspring is needed. Epigenetic ageing may be a mechanistic link between early-life exposures and risk of adult disease [191]. A recent study reported that a pro-inflammatory, immunological state during pregnancy was associated with shorter offspring telomere length [192]. Telomere biology plays a fundamental role in maintaining genome and cell integrity, and shortened telomere is a well-established indicator of the ageing processes [193]. Further research elucidating such mechanisms is required.

In conclusion, improving our understanding of parental nutritional programming may help inform the development of more effective public health messages, diet and lifestyle interventions and pregnancy guidelines to promote intergenerational health and longevity. Several studies have shown that dietary quality and dietary inflammation state during pregnancy play a role in child development, even if results from some studies remain inconsistent or limited. Strategies aiming to promote adherence to a less pro-inflammatory and better-quality diet across all stages of maternal and childhood life may be of considerable importance to public health.

## Figures and Tables

**Table 1:** Summary of Dietary Metric Components.

Dietary Score	Food Components and Calculation	Interpretation	Dietary Assessment Method
Dietary Inflammatory Index (DII) [30]	Literature-based index measuring the effect of 45 food parameters (a mix of macro- and micro-nutrient food components such as alcohol; vitamin B12; β-carotene; caffeine; carbohydrate; cholesterol; fibre; folic acid; garlic; ginger; iron; monounsaturated fat) on six inflammatory biomarkers (IL-1β, IL-4, IL-6, IL-10, TNF-α and C-reactive protein).	Higher values of the DII indicate a pro-inflammatory (i.e., less healthy) dietary profile, whereas lower values indicate an anti-inflammatory (i.e., more healthy) dietary profile. Using the energy-adjusted DII (E-DII) score is often more reliable as it is associated with improved prediction in comparison to unadjusted DII scores.	Food Frequency Questionnaire or 24-h recall
Mediterranean Diet (MD) [54,55]	Varying food components across different indices. Most useful indices base calculation of 9 components: - High intake of the non-Mediterranean foods: dairy and meat were scored negative as (0) - High intake of Mediterranean foods: cereals, legumes, fruit, vegetables, fish, Monounsaturated fatty acids to Saturated fatty acids (MUFA-SFA) ratio, and wine which were scored positive as (1)	A metric indicating compliance to the Mediterranean diet pattern in adults. Across the different MD indices, ranges calculated that generate higher values indicate greater MD adherence. A score of 0–3 represents low adherence, a score of 4–6 represents moderate adherence and a score of 7–9 represents high adherence to the Mediterranean diet	Food Frequency Questionnaire
Healthy Eating Index 2010 (HEI-2010) [56]	12 components in total 9 adequacy components: Total Fruit, Whole Fruit (forms other than juice), Total Vegetables, Greens and Beans (dark-green vegetables and beans and peas), Whole Grains, Dairy (all milk products and soy beverages), Total Protein Foods, Seafood and Plant Proteins, and Fatty Acids (ratio of polyunsaturated fatty acids [PUFA] and MUFA to SFA). Scored 10 in the highest consumption and 0 in the lowest. 3 moderation components are: Refined Grains, Sodium, and Empty Calories (all calories from solid fats & added sugars plus calories from alcohol beyond a moderate level) scored 10 in the lowest consumption	Measure of overall diet quality that measures alignment with the 2010 Dietary Guidelines for Americans Scored on a density basis out of 1000 calories An HEI score between 51 and 80 is considered as “needing dietary improvement”	Food Frequency Questionnaire
HEI-2015 [57]	13 components in total The HEI-2015 components are the same as in the HEI-2010, except Saturated Fat and Added Sugars replace Empty Calories. Previous versions of the HEI accounted for legumes in either the two vegetable or the two protein foods components, whereas HEI-2015 counts legumes toward all four components.	Measure of overall diet quality that measures alignment with the updated 2015–2020 Dietary Guidelines for Americans that are scored on a density basis out of 1000 calories. A HEI score between 51 and 80 is considered as “needing dietary improvement”	Food Frequency Questionnaire
AHEI-2010 [58,59]	11 components in total: Vegetables; fruit; whole grains; sugar-sweetened beverages; nuts and legumes; red and processed meat; trans fats; long-chain (n-3) fatty acids (eicosapentaenoic acid and docosahexaenoic acid); polyunsaturated fatty acid; sodium; alcohol	An alternative version of the HEI that focuses on adherence to a dietary pattern associated with chronic disease risk and pays more attention to fat quality (i.e., intakes of omega-3 fats and polyunsaturated fats) Assess diet adequacy and moderation	Food Frequency Questionnaire
Dietary Approaches to Stop Hypertension (DASH) [60,61]	8 components: Fruit, vegetables, nuts and legumes, whole grains, low-fat dairy products, sodium, red and processed meats, and sweetened beverages The lowest intake of healthy components (fruits, vegetables, nuts and legumes, low-fat dairy products, and whole grains) receive one point and the top quintile receives 5 points. The scoring for the remaining unhealthy components is reversely coded so that quintile 1 receives 5 points and quintile 5 receives one point	Sex-specific intake quintiles are generated for each of the 8 components. The overall score ranges from 8 (the lowest adherence) to 40 (the highest adherence)	Food Frequency Questionnaire
Glycaemic index and load (GI and GL) [62]	GI is a way of ranking carbohydrate foods based on the rate at which they raise blood glucose levels. GL estimates the impact of carbohydrate intake using the GI while accounting for serving size. GL = GI × carbohydrate in a serving/100	Foods that are digested quickly (e.g., refined, starchy grains and fruit juices) will raise blood glucose levels quickly and therefore are given higher GI/GL values A GL classification system is used in which foods are categorized as having low (≤10), medium (>10–<20) or high GL (≥20).	Food Frequency Questionnaire

**Table 2:** Individual studies on the associations between maternal dietary inflammation determined by the DII and E-DII and birth outcomes.

First Author, Year	Country or Setting	Study Design	Population	Dietary Assessment Method and Period	Assessment of Adherence to the Diet	Birth Outcomes	Key Findings
Navarro et al., 2019 [24]	Ireland, 2001–2003	Lifeways Cross-Generation Cohort Study: A prospective family study	1082 mother-child pairs	Semi-quantitative Food frequency questionnaires (FFQ), consumption during the first 12–16 weeks of pregnancy	Calculation of E-DII score based on 28 food parameters: carbohydrate, protein, fat, alcohol, fibre, cholesterol, saturated fat, mono-unsaturated fat, poly-unsaturated fat, niacin, thiamin, riboflavin, vitamin B12, vitamin B6, iron, magnesium, zinc, selenium, beta-caro- tene, vitamin A, vitamin C, vitamin D, vitamin E, folic acid, onion, garlic, tea and caffeine.	Low birthweight, macrosomia, per-term and post-term birth	Higher maternal E-DII scores were associated with increased risk of low birthweight Higher maternal grandmothers E-DII scores were associated with increased risk of macrosomia
Moore et al., 2018 [86]	USA, 2009–2014	Healthy Start study: A prospective pre-birth cohort.	1078 mother-child-pairs with neonates born ≥32 weeks of gestation, and mothers with pre-pregnancy BMI ≥18.5 kg/m2	Repeated 24-h dietary recalls completed each month during pregnancy	DII scores based on 28 components: energy, total fat, saturated fat, monounsaturated fat, polyunsaturated fat, omega-3 polyunsaturated fatty acids, omega-6 fatty acids, trans-fat, carbohydrates, fiber, protein, cholesterol, iron, Vitamin A, Vitamin C, Vitamin D, Vitamin E, niacin, thiamin, riboflavin, Vitamin B6, Vitamin B12, folic acid, magnesium, zinc, selenium, alcohol and caffeine	Birth weight, fat mass, fat-free mass and percent fat mass, small and large for gestational age (LGA)	Among neonates born to obese women, each one-unit increase in DII was associated with increased birth weight, fat mass and percent fat mass One-unit increase in DII score was associated with a tendency of increased risk of having a LGA infant
McCullough et al., 2017 [14]	USA, 2009–2011	NEST study: A prospective cohort study of pregnant women	1057 mother–child pairs with singleton deliveries	FFQs were administered at different time points: (1) at enrolment with a description of the diet during the periconceptional period; (2) during the second trimester to estimate usual diet in the first trimester; (3) between 36- week gestation and delivery date to estimate diet in the last 2 trimesters of pregnancy; and (4) at delivery	Calculation of E-DII score based on 27 food parameters: Carbohydrates, proteins, fats, alcohol, fibres, cholesterol, saturated fatty acids, monounsaturated fatty acids, polyunsaturated fatty acids, omega 3, omega 6, niacin, thiamin, riboflavin, vitamin B6, vitamin B12, iron, magnesium, zinc, selenium, vitamin A, vitamin C, vitamin D, vitamin E, folic acid, β carotene and caffeine	Sex-specific percentiles of birthweight for gestational age, small and large for gestational age, gestational age, mode of delivery	Women with pro-inflammatory diets had elevated rates of preterm birth among female offspring No association between infant birthweight and maternal E-DII score was observed
Sen et al., 2016 [15]	USA, 1999–2002	Project Viva, a pre–birth cohort study	1808 mother–child pairs with a pre-pregnancy BMI (in kg/m^2^) ≥18.5 without pre-existing type 1 or 2 diabetes mellitus	FFQ administered at the first and second trimester	DII based on 28 components: energy, carbohydrate, protein, fat, alcohol, fibre, cholesterol, SFAs, MUFAs, PUFAs, n–3 and n–6 FAs, *trans*-fat, niacin, thiamin, riboflavin, vitamin B-12, vitamin B-6, iron, magnesium, zinc, selenium, vitamin A, vitamin C, vitamin D, vitamin E, folic acid and β-carotene.	Birth weight for gestational age, premature birth	Higher DII scores were associated with lower birth weight for gestational age *z* score in infants born to obese mothers

**Table 3:** Individual studies on the associations between maternal dietary quality determined by the Mediterranean diet score and birth outcomes.

First Author, Year	Country or Setting	Study Design	Population	Dietary Assessment Method and Period	Assessment of Adherence to the Diet	Birth Outcomes	Key Findings
Yisahak et al., 2021 [92]	USA, 2009–2013	Eunice Kennedy Shriver National Institute of Child Health and Human Development Fetal Growth Studies-Singletons: Prospective cohort	2802 pregnant women	FFQ in first trimester	Trichopoulou’s score [71]	Birthweight, length, upper arm length, head circumference, abdominal circumference, sum of skinfold thick-ness, prematurity, SGA, LGA, low birth weight (LBW), macrosomia	Greater MD is associated with higher birthweight, reduced odds of LBW, increased birth length, upper arm length, significant p-trend for sum of skinfolds
Peraita-Costa et al., 2020 [93]	Spain, 2018–2019	Two-phase retrospective population-based study	1118 mother–child pairs admitted after delivery	Semi-quantitative FFQ (16 items), self-administered after delivery	Kidmex scores [72]	SGA and preterm birth	Medium adherence to MD was associated with a higher risk of giving birth to a preterm newborn. No association was found between MD adherence and SGA
Martinez-Galiano et al., 2018 [94]	Spain, 2012–2015	Prospective multicentre matched case-control study (The matching criterion was the maternal age at delivery)	518 mothers of singleton SGA infants, 518 mothers of singleton infants with normal weight for gestational age	Semi-quantitative FFQ, administered 2 days after delivery	PREDIMED score [95], Trichopoulou’s score [71], Panagiotakos’ score [96]	Small for gestational-age (SGA) risk	MD adherence was associated to a reduced risk of SGA in newborns
Gomez-Roig et al., 2017 [97]	Spain, over 14 months	Cross-sectional study	46 mothers with SGA foetuses, 81 mothers with appropriate for gestational age (AGA) foetuses	Semi-quantitative FFQ, administered during the third trimester	Trichopoulou’s score [71]	SGA risk	Mothers maintaining a Mediterranean-type diet were more likely to have an AGA foetus
Saunders et al., 2014 [90]	French Caribbean Island (Guadeloupe), 2004–2007	TIMOUN Study: Prospective mother–child cohort study	728 pregnant women with a live-born singleton pregnancy without major congenital malformations	Semi-quantitative FFQ, diet during pregnancy	Trichopoulou’s score [71]	Preterm delivery and risk of foetal growth restriction (FGR)	No association between MD adherence during pregnancy and the risk of prematurity or FGR MD adherence was associated with a decreased risk of prematurity specifically in overweight and obese women
Timmermans et al., 2012 [88]	Netherlands, 2001–2006	Generation R study, prospective population-based cohort study	3207 mothers with a spontaneously conceived live-born singleton pregnancy	Semi quantitative FFQ, Early pregnancy < 18 weeks	Dietary pattern identified and labelled MD as it was characterized by higher intakes of pasta, rice, vegetable oils, fish, vegetables and alcohol, and lower intakes of meat, potatoes and fatty sauces	Foetal growth, placenta development	Low MD adherence resulted associated with lower birth weight and placental weight
Chatzi et al., 2011 [89]	Spain 2004-2007, Greece 2007–2008	INMA (Spain) and RHEA (Crete) cohorts: Prospective population-based cohort study	Spain: 2461 mother–newborn pairs. Greece: 889 mother–newborn pairs	Semi-quantitative FFQ, administered during first (IMNA cohort) or mid trimester (RHEA cohort) of pregnancy	Trichopoulou’s score modified for pregnancy [71]	Foetal growth	INMA-Mediterranean cohort: high MD adherence was associated with higher BW and reduced risk of foetal growth restriction In all cohorts, high MD increased birth weight in smoking mothers
Mikkelsen et al., 2010 [91]	Denmark, 1996–2002	Danish National Birth Cohort: prospective cohort study	35657 pregnant women with a live-born singleton pregnancy	Semi-quantitative FFQ, self-administered at mid pregnancy	Khoury’s score [74]	Preterm delivery	Reduced risk of early preterm delivery in MD women
Haugen et al., 2008 [73]	Norway, 2002–2005	Norwegian Mother and Child Cohort Study (MoBa): prospective cohort study	26563 pregnant women with a live-born singleton pregnancy	Semi quantitative FFQ, administered at mid pregnancy	Khoury’s score [74]	Preterm delivery	No association with reduced risk of preterm birth

Trichopoulou’s score: Nine components including beneficial components (vegetables, legumes, fruits and nuts, cereal and fish), detrimental components (meat, dairy products, alcohol consumption) and fat intake. In pregnancy, dairy products are not considered detrimental and alcohol consumption is not included. Mediterranean-diet score ranged from 0 to 9. Khoury’ score: 5 MD criteria in the current study were defined by the intake of ≥5 vegetables and fruit/d, ≥2 servings of fish/wk, the use of olive or canola oil for cooking and salad dressings, ≤2 servings of red meat/wk, and ≤2 cups of coffee/d. PREDIMED score: development of a short dietary intake questionnaire for the quantitative estimation of adherence to a cardioprotective Mediterranean diet. Vegetables, legumes, fruit, fish, red and processed meat, chicken or poultry, olive oil for cooking, consumption of olive oil, butter-margarine, carbonated and/or sweetened beverages, commercial pastries, nuts and meals with sofrito (traditional sauce of tomatoes, garlic, onion or pepper in olive oil). The total score ranged from 0 (minimum adherence) to 14 (maximum adherence). Kidmex score: Based on the principles sustaining MD patterns as well as those that undermine it. Foods positively associated with the MD pattern, such as vegetables, legumes, fruits, nuts, cereal, fish, dairy products and olive oil, were assigned a value of +1, whereas foods with a negative association such as sweets and fast foods were assigned a value of –1. The score ranged from 0 to 12.

**Table 4:** Individual studies on the associations between maternal dietary quality determined by the Healthy Eating Index and birth outcomes.

First Author, Year	Country or Setting	Study Design	Population	Dietary Assessment Method and Period	Assessment of Adherence to the Diet	Birth Outcomes	Key Findings
Zhu et al., 2019 [103]	United States, 2014–2017	Pregnancy Environment and Lifestyle Study: A prospective cohort	2269 multi-racial/ethnic women	FFQ during early pregnancy (10–13 weeks) that collected information on habitual dietary intake during the previous 3 months.	HEI-2010 [64] Exclusion of alcohol consumption	Birthweight z-score, large- and small-for-gestational age	79% of pregnant women did not adhere to the Dietary Guidelines for Americans. Poor diet quality in pregnancy was associated with higher birthweight and increased risk of LGA independent of maternal obesity and other covariates
Navarro et al., 2019 [104]	Ireland, 2001–2003	Lifeways cross generation cohort study: prospective family study	1082 families at birth	Semi quantitative FFQ during the first trimester of pregnancy	The HEI-2015 [57]	Birth weight, macrosomia, premature birth	Higher scores on the maternal HEI were associated with lower risk of low birth weight. Similar associations were found for maternal grandmothers with higher HEI scores, but a slightly risk of macrosomia was observed
Chia et al., 2018 [105]	Singapore, 2009–2010	The GUSTO Study, a prospective mother-offspring cohort	1051 pregnant women	24-h recalls at 26–28 weeks of gestation	They used the HEI-SGP (11 components with a maximum possible raw score of 90) *	Preterm birth, offspring birth size, and adiposity	Higher maternal diet quality during pregnancy was associated with longer birth length and lower neonatal adiposity No associations with birth weight or preterm birth
Badon et al., 2017 [100]	United States, 1996–2008	Omega study, a prospective pregnancy cohort	2924 women with singleton live births	Self-administered semi-quantitative FFQ, diet over the last 3 months	AHEI-2010 [65] Alcohol consumption was not included	Birthweight, large- and small-for-gestational age	No associations were found with birth anthropometry
Shapiro et al., 2016 [102]	United States, 2014	The Healthy Start cohort, a pre-birth observational cohort	1079 singleton mother-offspring pairs, without prior medical history	Self-Administered 24-h dietary recalls during pregnancy	HEI-2010 [64] Exclusion of alcohol consumption	Neonatal adiposity	Having an HEI-2010 score ≤57 (poorer diet quality) was significantly associated with higher %fat mass
Poon et al., 2013 [101]	US, 2005–2007	The Infant Feeding Practices Study II, a prospective cohort study	830 women included for analyses after exclusion of women with diabetes	Self-completed Diet History Questionnaire during the third trimester that reflects dietary intake between 28 and 36 weeks of gestation	Alternative Healthy Eating Index for Pregnancy (AHEI-P) updated from the updated AHEI-2010 alcohol was excluded, while calcium, folate, and iron were added to the scoring method	Birthweight z-scores and weight-for-length z-scores	Maternal diet quality was not associated with birth anthropometry
Rodriguez-Bernal et al., 2010 [99]	Spain, 2004 2005	The Valencia birth cohort—A subproject of the INMA study	787 women and their newborns	FFQ, administered by trained interviewers during the first trimester of pregnancy	AHEI adapted for pregnancy ** To make the index more appropriate for the pregnant population, they excluded the alcohol intake and long-term multivitamin use. They added folate, iron, and calcium intakes [106]	Birth weight, birth length, and head circumference at birth	Newborns of women in the fourth quintile were heavier and longer than those in the lowest quintile. Decreased risk of foetal growth-restricted infant for weight in women with the highest AHEI.
Rifas-Shiman et al., 2009 [75]	US, 1999–2002	Project Viva: prospective cohort study	1777 women with singleton live births	Self-administered semi-quantitative FFQ assessing the woman’s diet during early pregnancy and the second trimester	AHEI-P: Exclusion of alcohol, nuts and soy protein component and inclusion of tofu or soybeans in the vegetable component, folate, iron, and calcium	Birth weight for gestational age categories: small and large for gestational age	No associations with foetal growth

* HEI-SGP: Fruit and vegetables (0–20 points), rains (0–20 points), meat and others (0–20 points), total fat (0–10 points) and total saturated fat (0–10 points); and last, the use of antenatal supplements containing iron, folate and calcium (0–10 points).** AHEI consisted of intake of 9 items: Vegetables (5 servings/d),fruit (4 servings/d), nuts and soy (1 serving/d), ratio of white meat (fish and poultry) to red meat (≥4:1), cereal fibre (15 g/d),trans fat (≤0.5% of energy), ratio of polyunsaturated to saturated fat (≥1), moderate alcohol intake (0.5–1.5 servings/d) and long-term multivitamin use (≥5 y of continuous use).

**Table 5:** Individual studies on the associations between maternal DASH scores and birth outcomes.

First Author, Year	Country or Setting	Study Design	Population	Dietary Assessment Method/Intervention and Period	Assessment of Adherence to the Diet	Birth Outcomes	Key Findings
Jiang et al., 2019 [82]	China, 2015–2017	Randomized controlled Trial	85 pregnant women diagnosed with gestational or chronic hypertension	Intervention: DASH diet vs. control (usual diet), randomly assigned at 12 weeks	The Dietary Approaches to Stop Hypertension (DASH) diet was modified to accommodate specific needs of pregnancies. The diet was similar to the control diet in terms of macronutrients (50–60% carbohydrate, 20–25% fat and 20–25% protein); however, it was rich in fruits, vegetables, whole grains and low-fat dairy products; low in saturated fats, cholesterol and sweets. The amount of salt intake was 4g/d.	Prematurity, birth weight, body length and Apgar score	Beneficial effects on the incidence of prematurity, low birth weight and infant body length
Van Horn et al., 2018 [109]	USA, 2012–2015	Maternal Offspring Metabolics Family Intervention Trial (MOMFIT): Randomized controlled Trial	281 overweight/obese women randomized at 16-week gestation	Intervention DASH diet vs. usual care. The two groups received the 2008 Physical Activity Guidelines for Americans.Second and third trimester of pregnancy	Minor modifications to the DASH diet, called MAMA-DASH, were calorically suited to the restricted weight gain recommendations, and followed nutrition guidelines for pregnant women, including avoidance of fish considered higher in mercury, inclusion of calcium-rich, vitamin D−enriched dairy, or calcium-fortified non-dairy products	Newborn anthropometry	No significant differences in birth anthropometric measurements, percentage body fat
Fulay et al., 2018 [110]	USA, 1999–2002	Project VIVA: longitudinal cohort study	1760 women included with a singleton pregnancy	Semi-quantitative FFQ, at study enrolment in the first trimester of pregnancy	Calculation of two dietary patterns: DASH diet and DASH OMNI pattern, which is based on the original DASH diet supplemented by higher unsaturated fat intake	Birth size and preterm delivery	Adherence to neither DASH or DASH OMNI diet during early pregnancy were associated with prematurity or birth anthropometry
Vesco et al., 2015 [108]	USA, 2009–2011	Randomized controlled Trial	114 obese women randomized between 7–21 weeks’ gestation	DASH diet with least 30 min of moderate physical activity vs. control group who received one time advice session without any particular focus on DASH diet or weight management	Energy reduced eating plan, based on DASH dietary pattern without sodium restriction.	Small and large for gestational age, weight-for-gestational-age z-score, macrosomia	Intervention group had lower risk of having LGA infant
Martin et al., 2015 [111]	USA, 1995–2000	PIN (Pregnancy, Infection, and Nutrition): Prospective cohort study	3143 pregnant women included	Self-administered FFQ 26–29 weeks of gestation	DASH diet was characterized positively by intake of fruits, vegetables, nuts and legumes, low-fat dairy and whole grains. Sodium, red and processed meats, and sweetened beverage were reversed-scored	Premature birth	Greater adherence to the DASH diet is associated with decreased odds of prematurity compared with women in the lowest quartile
Asemi et al., 2014 [78]	Iran, 2013	Randomized controlled Trial	52 women diagnosed with gestational diabetes mellitus	Intervention: DASH diet for 4 weeks during pregnancy vs. control diet with similar calorie content and protein composition	DASH diet was rich in fruits, vegetables, whole grains and low-fat dairy products, and low in saturated fats, cholesterol, refined grains and sweets. The amount ofsodium intake was 2400 mg per day	Birthweight, Birth length, Head circumference at birth, Ponderal Index, Apgar score, Macrosomia, Polyhydramnios, Gestational age	Lower birth weight, head circumference and ponderal Index in the intervention group

**Table 6:** Individual studies on the associations between maternal glycaemic index and birth outcomes.

First Author, Year	Country or Setting	Study Design	Population	Dietary Assessment Method/Intervention and Period	Assessment of Adherence to the Diet	Birth Outcomes	Key Findings
Wahab et al., 2020 [35]	Netherlands, 2002–2006	Generation R: Population-based prospective birth cohort study	3471 pregnant women without any medical conditions	Semi-quantitative FFQ in first trimester	GI values were obtained from the glycaemic index database on the Dutch diet published by the Medical Research Council Human Nutrition Research Calculation of the GI and GL	Foetal growth in mid and late pregnancy Birth weight, risk of SGA, LGA, Preterm birth	Higher maternal early pregnancy GL was associated with a higher foetal abdominal circumference and estimated foetal weight in late pregnancy. Higher maternal early pregnancy GI was associated with a lower risk of a large-for-gestational-age infant
Chen et al., 2019 [38]	Ireland, 2001	Lifeways study: Prospective cohort study	842 mother–child pairs with singleton live births	Semi-quantitative FFQ, self-completed at the first 12–16 weeks of pregnancy	To calculate dietary GI and GL, food items listed in the FFQ were matched to corresponding food items in databases with published GI values. Average participant GL was derived by summing the food intake frequency-weighted GL for all food items. The average dietary GI of participants was calculated by dividing their GL by their total available carbohydrate intake, then multiplying by 100.	Birth weight, macrosomia, gestational age	Neither glycaemic nor insulinemic responses to diet during early pregnancy appear to be associated with offspring birth weight, gestational age, BMI at birth
Gomes et al., 2019 [40]	Brazil, 2012–2013	Cohort study	259 women	Two 24-h dietary recalls in each gestational trimester	The dietary GI and GL values were obtained from the Nutrition Data System for Research software.Calculated for the second and third trimester	Birth weight z-score calculated using the e Intergrowth-21st Project	No association between GI/GL and birth weight were observed
Horan et al., 2016 [113]	Ireland, 2007–2011	The ROLO study: Randomized controlled trial	280 mother-infant pairs. Participants were secundigravida women with a previous macrosomic baby (>4 kg).	Low GI dietary advice vs. usual antenatal care, which did not involve dietary advice. 3-day food diaries were completed at each trimester of pregnancy	GI values were determined using the 2008 International Tables of GI values and other recently published GI values	Offspring adiposity at 6 months	No difference was found in 6 months infant adiposity between control and intervention groupsbut maternal trimester 3 GI was positively associated with 6 months old triceps skinfold thickness for age z-score and biceps skinfold thickness
Moses et al., 2014 [114]	Australia, 2010	PREGGIO (Pregnancy and Glycaemic Index outcomes): A randomized controlled trial	576 women without any medical conditions	Low GI dietary advice at the first antenatal visit vs. healthy eating diet. Participants were provided with 1 of 2 sets of booklets (depending on their allocation) that included information on the choices for and serving sizes of carbohydrate-rich foods. No intended difference in the macronutrient distribution in diets	Database incorporated Australian food-composition tables and published GI valuesThe dietary GI was calculated as the weighted sum of the GI of all carbohydrate (CHO) foods in the diet, with the weighting proportional to the contribution of each food to total CHO intake. Glycaemic load is the product of the GI and amount of CHO.	Birth anthropometry and ponderal index	No significant differences in foetal birth weight, length, and ponderal index between the two groups. GL was the only predictor of birth percentile and ponderal index.
Knudsen et al., 2013 [83]	Denmark, 1996–2002	Danish national birth cohort: Prospective birth cohort study	41,782 first trimester pregnant women	Self-administered FFQ in week 25 of gestation	Values of the GI were extracted from published data and were incorporated into the dietary database. Calculation of the GI and GL	Birth weight, SGA or LGA risks	Mean of birthweight was higher in the highest GL quintile compared to the lowest and increased risk of LGA was observed in the highest GL quintile.
Walsh et al., 2012 [81]	Ireland, 2007–2011	The ROLO study: Randomized controlled trial	800 secundigravida women with a previous macrosomic baby (>4 kg).	Low GI diet from 14 weeks gestation to delivery vs. no dietary intervention. Research dietitian met the patients at 28 and 34 weeks’ gestation.	Women were encouraged to choose as many low GI foods as possible and to exchange high GI carbohydrates for low GI alternatives. Women were not advised to reduce their total caloric intake.	Birth weight	No significant difference was seen between the two groups in absolute birth weight, birth length, head circumference or ponderal index at birth
Perichart-Perera et al., 2012 [112]	Mexico, 2004–2008	Randomized controlled trial	107 women recruited with gestational diabetes or pregestational type 2 diabetes mellitus	Control group (*n* = 61): women received an individual food plan based on CHO restriction. Women in this group were advised to choose any type of CHO, except added refined sugars. Intervention group (*n* = 46): women were counselled to eliminate all moderate and high GI foods (GI > 55) Energy and CHO prescriptions were revised at every visit and changes were done according to weight gain	Educational themes included the importance of healthy eating with diabetes, identification of CHO, exchange lists and CHO counting, identification of high and low GI foods, healthy fats and importance of capillary glucose self-monitoring. Significant decrease in the GI in the interventional group	Birth anthropometry measurements, macrosomia, prematurity,	A trend of lower birthweight was observed in the intervention group without risk of SGA. Higher frequency of premature birth was observed in the intervention group.

**Table 7:** Studies investigating maternal dietary scores and long-term offspring outcomes.

First Author, Year	Country/Setting	Study Design	Population	Maternal Dietary Assessment Method	Assessment of Adherence to Diet/Dietary Score	Long-term Outcome/Median Child Age	Key Findings
Callanan et al., 2021 [115]	Ireland2007–2011	Randomized controlled trialThe ROLO study	387 mother–child pairs	3-day food diary during each trimester of pregnancyLow GI diet from 14 weeks gestation to delivery vs. no dietary intervention	Low eucaloric GI diet (dietary education sessions with a research dietitian) vs. control (routine antenatal care)	Anthropometry and child adiposity at 5 years	No significant associations were found between low GI in pregnancy RCT group and child weight/body composition outcomes (these included weight, BMI, centiles, waist–hip ratio, central adiposity, total fat mass and total lean mass) at 5 years follow-up.
Strohmaier et al., 2020 [116]	USA	Retrospective observational analysis of the Nurses’ Health Study II (NHSII) and Growing Up Today Study II (GUTSII) cohorts.	2729 mother–child pairs	FFQof habitual diet within last 12 months during peripregnancy period	AHEI-2010 [65], Alternate Mediterranean Diet (aMED) (Trichopoulou’s score modified for pregnancy) and DASH based on 8 components (fruits, vegetables, nuts and legumes, low-fat dairy and whole grains, sodium, red and processed meat and sugar-sweetened beverages)	Anthropometry-overweight and obesity, 12–23 years	Greater maternal adherence to aMED and DASH, but not AHEI, was associated with lower overweight risk in the offspring (RR_Q5 vs Q1_ = 0.82 for aMED and 0.86 for DASH, *P* for trend <0.05 for both) but none of the 3 scores remained significantly associated with offspring overweight or obesity risk after further adjustment for maternal pre-pregnancy BMI and lifestyle factors (maternal smoking status and physical activity) before pregnancy
Maslova et al., 2019 [117]	Denmark	Population-based cohort study-Danish National Birth Cohort (DNBC)	68,471 mother–offspring pairs	360-items semi-quantitative FFQ during mid-pregnancy (25-week gestation)	Reference of GI were retrieved from published data relevant to the time period ofenrolment Calculation of the GI and GL per day	Age- and sex-specific BMI *z* scores and growth velocities at 7 years	Higher maternal GI and GL were associated with an increase in the 7-year BMI *z* score in the unadjusted analysis; association was not significant in adjusted analysis. Children of underweight women showed a potential higher risk of overweight/obesity at age 7 with higher maternal GI and GL, but with wide confidence intervals.
Chen et al., 2019 [38]	Ireland	Prospective cohort-Lifeways Cross-Generation Cohort Study	842 mother–child pairs	FFQduring the first 12–16 weeks of pregnancy	Food items were matched to corresponding food items in databases with published GI values using a UK database. Calculation of the GI, GL, II and IL	Children’s body mass index (BMI) and waist circumference at 5 years	No association was observed for scores with BMI & waist circumference z-scores and childhood obesity (general and central) at 5-y follow-up
Gonzalez-Nahm et al., 2019 [118]	Southwestern USA	Prospective cohort-Nurture Study	623 mother–infant pairs	FFQ during 2nd-3rd trimester	AHEI-2010 [65] (excluding alcohol)	Anthropometry, at birth, 6 months, 12 months	After adjustment, maternal AHEI-2010 was not associated with infant adiposity at birth, 6 or 12 months.
Tahir et al., 2019 [119]	USA	Prospective cohort-MILK Study	354 mother–infant pairs	FFQ3rd trimester, 1 month postpartum, 3 months postpartum	HEI-2015 [57]	Anthropometry at birth, 1 month, 3 months and 6 months	A 10-unit increase in HEI–2015 total scores during pregnancy was associated with an approximately 0.6% lower infant body fat% at six months (β = −0.58, *p* = 0.05).
Navarro et al., 2019 [24]	Ireland	Prospective cohort-Lifeways Cross-Generation Cohort Study	1082 child–mother pairs; 585 children	FFQduring the first12–16 weeks of pregnancy	E-DII (as described in Table 2)	Anthropometric Outcomes 5 years and 9 years	No associations were found between maternal E-DII scores and offspring overweight and/or obesity at 5 and 9 years
Navarro et al., 2019 [104]	Ireland	Prospective cohort-Lifeways Cross-Generation Cohort Study	1082 child–mother pairs; 585 children	FFQduring the first 12–16 weeks of pregnancy	HEI-2015 [57]	Anthropometric Outcomes at 5 years	No associations between maternal HEI scores and childhood overweight or obesity; higher paternal HEI scores were associated with lower odds ratios of childhood obesity at 5 years old
Sen et al., 2018 [84]	USA	Longitudinal cohort-Project Viva	922 mother–child pairs	Validated semi quantitative FFQ completed between 10-28-week gestation	DII calculated based on 28 dietary parameters: energy, carbohydrate, protein, fat, alcohol, fibre, cholesterol, SFAs, MUFAs, PUFAs, n–3 and n–6 FAs, *trans*-fat, niacin, thiamin, riboflavin, vitamin B-12, vitamin B-6, iron, magnesium, zinc, selenium, vitamin A, vitamin C, vitamin D, vitamin E, folic acid, and β-carotene.	Anthropometric Outcomes (range 6–10 years, median 7.7 years)	Higher maternal DII in pregnancy was directly associated with offspring size in mid-childhood, including BMI z-score, fat-free mass index and waist circumference as well as more direct measures of adiposity and dysmetabolism, including fat mass index, trunk fat mass index and fasting insulin
Chatzi et al., 2017 [120]	USA and Greece	Population-based prospective cohorts	1566 mother–child pairs	FFQ during 1st trimester	MDS ranging from 0–9, Trichopolous score modified based on recommendations for pregnant women [71]	Child adiposity, blood pressure and cardiometabolic parameters Mid-childhood (median 7.7 years USA; median 4.2 years Greece)	For each 3-pt increment in the MDS: offspring BMI *z*-score was lower by 0.14 units (95% CI, −0.15 to −0.13), waist circumference by 0.39 cm (95% CI, −0.64 to −0.14), the sum of skin-fold thicknesses by 0.63 mm (95% CI, −0.98 to −0.28). lower offspring systolic (−1.03 mmHg; 95% CI, −1.65 to −0.42) and diastolic blood pressure
Fernandez-Barres et al., 2016 [121]	Spain	Population-based ‘Infancia y Medio Ambiente’ (INMA) birth cohort study	1827 mother–child pairs	FFQ during 1st and 3rd trimester	Relative Mediterranean diet score (rMED), final potential score range was 0–16	Anthropometry-Overweight/obesity and abdominal obesity based on waist circumference at 4 years	Higher adherence to the MD in pregnancy was not associated with offspring overweight at age 4 years, but there was some evidence of an inverse, moderate association between maternal rMED scores in pregnancy and offspring waist circumference
Okubo et al., 2014 [122]	UK	Prospective cohort – Southampton Women’s Survey	906 mother–child pairs	FFQBetween 11–34-week gestation	GI and GL GI values were obtained from the GI database published by the Medical Research Council Human Nutrition Research, UK The GL of each food item was determined by multiplying the carbohydrate content of one serving by its GI value (divided by 100)	Body fat mass and lean mass, 4 years and 6 years	Maternal dietary GI in early (11 weeks) pregnancy was positively associated with fat mass at 4 and 6 years of age. Maternal dietary GI in late (34 weeks) pregnancy was also positively associated with fat mass at 4 and 6 y of age, but these associations were not robust to adjustment for confounding factors

## Data Availability

Not applicable.

## Abbreviations

AA	Amino acids
AGA	Appropriate for gestational age
AHEI	Alternative Healthy Eating Index
AHEI-P	Alternate Healthy Eating Index for Pregnancy
ALSPAC	Avon Longitudinal Study of Parents and Children
aMED	Alternate Mediterranean Diet
BMI	Body mass index
BP	Blood pressure
CHO	Carbohydrate
CVD	Cardiovascular disease
DASH	Dietary Approaches to Stop Hypertension
DHA	Docosahexaenoic acid
DII	Dietary inflammatory index
DNBC	Danish National Birth Cohort
E-DII	Energy-adjusted DII
EPA	Eicosapentaenoic acid
FA	Fatty acids
FMI	Fat mass index
FFMI	Fat-free mass index
FFQs	Food frequency questionnaires
FGR	Foetal growth restriction
GDM	Gestational diabetes mellitus
GI	Glycaemic Index
GL	Glycaemic Load
HEI	Healthy Eating Index
HF	High-fat
*IGF2*	Insulin-like growth factor 2
II	Insulinemic index
IL	Insulinemic load
INMA	Infancia y Medio Ambiente birth cohort study
IUGR	Intrauterine growth retardation
LBW	Low birth weight
LGA	Large for gestational age
*LINE-1*	Interspersed nuclear element 1
MD	Mediterranean diet
MDS	MD Score
MDScale	Mediterranean Diet Scale
MEDLIFE	Mediterranean Lifestyle
MetS	Metabolic syndrome
MFP	Mediterranean Food Pattern index
NEST	Newborn Epigenetics study
NTD	Neural tube defects
OCM	One-carbon metabolism
OWOB	Overweight and obesity
*POMC*	Proopiomelanocortin
PUFA	Polyunsaturated fatty acids
RCTs	Randomised controlled trials
rMED	Relative Mediterranean diet score
SGA	Small for gestational age
*TAPBP*	TAP Binding Protein gene
T2DM	Type 2 diabetes mellitus
UmPI	Umbilical artery pulsatility index
UPBEAT	UK Pregnancies Better Eating and Activity Trial
USDA	United States Department of Agriculture.
